# Planning Engagement With Web Resources to Improve Diet Quality and Break Up Sedentary Time for Home-Working Employees: A Mixed Methods Study

**DOI:** 10.1037/ocp0000356

**Published:** 2023-08

**Authors:** Dawn Holford, Gianluca Tognon, Valerie Gladwell, Kelly Murray, Mark Nicoll, Angela Knox, Rachel McCloy, Vanessa Loaiza

**Affiliations:** 1 Department of Psychology, University of Essex; 2 School of Psychological Science, University of Bristol; 3 School of Health Sciences, University of Skövde; 4 School of Sport, Rehabilitation and Exercise Sciences, University of Essex; 5 Institute of Health and Wellbeing, University of Suffolk; 6 Keep Fit Eat Fit Wellbeing Ltd, Chelmsford, United Kingdom; 7 School of Psychology and Clinical Language Sciences, University of Reading

**Keywords:** action planning, sedentary behaviour, dietary quality, working from home, online intervention

## Abstract

As home working becomes more common, employers may struggle to provide health promotion interventions that can successfully bridge the gap between employees’ intentions to engage in healthier behaviors and actual action. Based on past evidence that action planning can successfully encourage the adoption of healthier behaviors, this mixed-methods study of a web-based self-help intervention incorporated a randomized planning trial that included quantitative measures of engagement and follow-up qualitative interviews with a subsample of participants. Participants either (a) selected a movement plan for incorporating a series of 2-min exercise videos into their work week to break up sedentary time and a balanced meal plan with recipe cards for a week’s lunches and dinners or (b) received access to these resources without a plan. Selecting a movement plan was more effective at increasing engagement with the web resources compared to the no-plan condition. In the follow-up interviews, participants indicated that the plan helped to remind participants to engage with the resources and made it simpler for them to follow the guidance for exercises and meals. Ease of use and being able to fit exercises and meals around work tasks were key factors that facilitated uptake of the resources, while lack of time and worries about how colleagues would perceive them taking breaks to use the resources were barriers to uptake. Participants’ self-efficacy was associated with general resource use but not plan adherence. Overall, including plans with online self-help resources could enhance their uptake.

Sedentary behavior and poor dietary habits contribute jointly to obesity-related chronic health conditions, which can cost employers billions every year in lost productivity (Goettler et al., 2017; Hall et al., 2021) and are associated with higher absenteeism and lower work performance (Abraham & Graham-Rowe, 2009; Fitzgerald et al., 2016). Unfortunately, recent measures to combat the spread of the COVID-19 pandemic, which required unprecedented numbers of employees to work from home, have had negative effects on lifestyle behaviors including increases in sitting time (Meyer et al., 2020; Ráthonyi et al., 2021) and poorer diet quality (Mattioli et al., 2020; Oni et al., 2020; Robinson et al., 2021). There is a concern that these trends may continue postpandemic as more workplaces transition, many permanently, to remote working (Bartmann et al., 2023; Chavez-Dreyfuss, 2020; Peters et al., 2022).

In light of these worrying trends, interventions that can break up sitting time with home-based physical activity and improve dietary quality are strongly recommended (Mattioli et al., 2020; Parekh & Deierlein, 2020). However, interventions that focus on changes in the office environment (e.g., providing height-adjustable desks: Munir et al., 2018; offering healthy food in cafeterias: Hendren & Logomarsino, 2017) have had modest improvements on these outcomes but are difficult to deliver if employees are no longer on site (Bartmann et al., 2023). Therefore, interventions that can be delivered online could be more feasible and reach more employees. These could be done using wearable devices to prompt and measure physical activity (e.g., activity trackers, smart watches; Macridis et al., 2018; Xie et al., 2018), but these devices are not affordable for all employers or employees, and there is a growing concern about privacy with employer-provided health-monitoring devices (Binyamin & Hoque, 2020; Richter, 2020). Web-based health interventions that can rely on general computing equipment (e.g., smartphones, laptops) that the home worker would already possess are more accessible to organizations and can reach a wider number of employees. These tend to be informational in nature, for example, sending employees information about the benefits of healthy diets and exercises (Anderson et al., 2009).

Unfortunately, many of these interventions, such as those that targeted general information provision and advice for health behavior, have had limited success compared to interventions that included in-person components, such as behavioral counseling (Anderson et al., 2009). This is possibly because they tended to target a single aspect of behavioral change (increasing knowledge) without addressing the barriers to putting that knowledge into practice. Larger and more consistent effects could be achieved by drawing on other factors identified by theories of behavioral change (e.g., Michie et al., 2020). In this study, we investigate these factors in the context of an intervention to help home-based employees decrease sedentary time and prepare balanced meals.

## Theoretical Framework for Behavior Change

Behavioral change is a multifaceted process affected by many different factors. For example, personality factors (e.g., conscientiousness), social support, the value placed on the behavior, stress, health literacy (e.g., education and knowledge about the importance of health behaviors), information processing ability, and environmental factors (e.g., access to health care), all contribute to an individual’s likelihood of adhering to an intervention program (Conner et al., 2007; Eynon et al., 2019; Marcus et al., 1996; Michie et al., 2008). Behavioral change models consider the relative importance of these factors and how they interact to produce change. For instance, the theory of planned behavior (Ajzen, 1991) posits that intention to change and perceived level of control over the behavior contribute to actual behavioral change, and factors such as attitudes and social norms affect these two drivers of change (e.g., McDermott et al., 2015; McEachan et al., 2011). The capability, opportunity, motivation–behaviour (COM-B) model, derived from a meta-analysis of behavioral change intervention frameworks (Michie et al., 2009, 2011), proposes that engaging in a behavior is supported by an individual’s “Capability” to engage in it (i.e., their physical and psychological capacity to undertake the behavior), “Opportunity” to do so (i.e., external factors that prompt or facilitate the behavior, including social or cultural norms and environmental or logistical facilitation), and “Motivation” to perform it (i.e., cognitive processes that drive behavior, including those that assist with planning or habituating behavior). Within the model, the different components are interlinked, such that effecting change in one can influence behavior directly and also indirectly by affecting another component. For example, giving people recipes to try can increase their capability to make a healthy meal, while also relieving the cognitive burden (i.e., lack of motivation) of having to overcome their lack of capability.

Behavior change frameworks are particularly helpful in understanding why people who value a behavior and intend to change still struggle to put these actions into practice (Orbell & Sheeran, 1998; Sniehotta et al., 2005; Tuman & Moyer, 2019; Webb & Sheeran, 2006). For example, one can intend to break up one’s sitting time throughout the work day, but fail to do so because colleagues have scheduled multiple online meetings with only short breaks. This reason can be understood as a lack of perceived control and supportive social norms (theory of planned behavior), and decomposed into the lack of capability (inadequate knowledge of how to utilize short breaks), opportunity (no prompts to move during the short breaks) and motivation (no plan to deal with the challenges posed by the meeting schedule; COM-B). Conceptualizing the antecedents of behavioral change as an interlinked system of distinct components means that interventions may target one or more components to help these individuals address their “intention-behavior gap” (Michie et al., 2011). In the case of someone who wants to reduce sedentariness, a helpful intervention would thus be one that increases their capacity, creates opportunities, and facilitates motivational processes to achieve the goal.

One way to target the components that help to convert one’s desire for change into actual change is to formulate a plan. Studies on plan interventions report robust results ranging from Cohen’s *d* = 0.3–0.5 (Bélanger-Gravel et al., 2013; Luszczynska et al., 2011; Olander et al., 2013) in various domains such as healthier eating (Verplanken & Faes, 1999) and exercising (Bélanger-Gravel et al., 2013; Sniehotta et al., 2005). A plan specifies the action to be taken (e.g., do some exercises) at a given time (e.g., when one has a gap between meetings), thereby providing a road map for how to reach the intended goal. In the context of the COM-B model, a plan directly affects the motivation component by simplifying the cognitions involved in engaging in behavior, but it could further maximize the chance of success if it also includes resources that improve individuals’ capability to perform the behavior (e.g., providing the exact exercises to perform) and help them to structure their environment to provide opportunities for it (e.g., prompting the individual to do their exercises).

## An Online Intervention to Improve Diet Quality and Break Up Sedentary Time

In this mixed-methods study, our objective was to identify factors that might facilitate employees’ engagement with an online health intervention that encouraged them to improve their diets (by trying simple, balanced recipes) and reduce chronic sedentary behavior (by doing short exercises at regular intervals throughout a standard 8-hr working day). We focused on these two behaviors as they hold benefits for almost all individuals whose work involves long periods of sitting, even if those individuals are also physically active (Healy et al., 2010; Koster et al., 2012; Owen et al., 2010).

The basic intervention focused on building capability and opportunity through providing online resources to participants and prompts to use them. We sought to identify facilitators and barriers to using the web resources through all participants’ qualitative answers to questionnaires and poststudy interviews with a subsample of participants. In addition, building on the evidence base that planning one’s actions should better encourage behavioral change by simplifying cognitive processes involved in initiating the behaviors (i.e., “motivation”), we included an embedded randomized trial in which participants either received a plan for how to use the web resources or they did not receive any plan. We hypothesized that a plan (compared to no plan) would increase the likelihood of participants using the resources and their self-efficacy in improving diet and overcoming sedentariness. We also hypothesized that participants’ baseline self-efficacy (as a marker of existing capability and motivation) would be positively correlated with greater resource use and plan adherence.

## Method

The project received ethical approval from the University of Essex’s ethics committee. The study hypotheses, design, methods, and analyses were preregistered prior to the data collection and are available on the open science framework (OSF; https://osf.io/dbk6z/). The data that support the findings of this study and the analysis code are available on the OSF and UK Data Archive (https://doi.org/10.5255/UKDA-SN-855578). The study was conducted in collaboration with a UK company, Keep Fit Eat Fit Wellbeing Ltd (KFEF), which provided the web resources used in the project.

### Participants

The final sample included 67 participants (85% female, age range = 25–64 years, *M* = 39.46, *SD* = 9.75) from a large organization in the higher education sector that was a prospective client of KFEF. Because the study was conducted as an industry collaboration, our sample size was determined as a matter of practicality with a target of at least 60 participants based on what the company could achieve within the funding time frame. We conducted sensitivity analyses that determined the sample size gave a chance to detect a large effect (*d* = 0.76, α = .05, 1 − β = 0.90) in a two-group comparison for the quantitative portion of the study. For a medium effect (*d* = 0.5), the achieved power was 65%.

The attrition rate was 17% (14 dropouts out of 81 total recruited participants). To ensure that participants were from a group for whom breaking up sitting time would be beneficial (regardless of existing physical activity), we only included participants who reported that they typically spent over 4.8 hr of their working day sitting.^1^ Participants’ median and mean work from home days and mean hours spent sitting are reported in Table 1, along with other demographic and prestudy measures. Our sample was comparable to recent research in terms of their sedentariness: on average 7.25 hr, versus 7.47 hr reported in a study of 317 UK employees in a similar work setting to ours (Faghy et al., 2022).[Table tbl1]

### Design

The study used a mixed-methods approach that incorporated a randomized two-group intervention with quantitative measures, along with a poststudy feasibility analysis and follow-up qualitative interviews with a subsample of 10 participants (50% female,^2^ 50% from each intervention condition, details in Table 2). Participants were randomly assigned by computer software to either select a meal and movement plan for the study period (*n* = 33) or to the no-plan control condition (*n* = 34).[Table tbl2]

### Procedure

We describe here the procedure in brief, and the precise details of how each part was operationalized are provided in Supplemental Material and on the OSF. Participants signed up to the study and were given a free membership login to KFEF’s web portal at least 10 days prior to the 5-day period (Monday to Friday) in which they would complete the study, so that they could familiarize themselves with the platform, be sent study materials, and make any preparations needed (e.g., purchase recommended ingredients). As part of the sign-up process, participants gave informed consent and completed an initial questionnaire including prestudy measures (see Materials and Measures section) and demographic information (see Table 1). Participants were randomized to the plan or no-plan control condition, and those in the plan condition selected one of three prepared plans for their meals and daily movements, which was sent to them before the study.

During the study period, participants received daily email reminders from the KFEF email system to use the exercise videos (at 9 am) and recipe cards (at 11 am). The emails contained links to where these resources were found on the web portal for participants in the no-plan condition. For participants in the plan condition, the emails contained direct links to the resources in their plan. Participants also received email reminders on Monday, Wednesday, and Friday to fill in a Daily Food List online.

On the day after their study period completed, participants received by email the link to a poststudy questionnaire and were given a week to complete this, with regular reminders sent if they had not yet completed it. Participants who completed the entire study were offered £25 in shopping vouchers.

### Materials and Measures

#### Recipe Cards

A set of 30 recipes were designed by the second author (who is a trained nutritionist) according to two objectives: (a) providing a balanced contribution to advised dietary targets (e.g., Public Health England, 2018) and (b) lowering barriers (e.g., time, cost) for participants by ensuring meals were easy to prepare (e.g., containing no more than seven common, easily accessible main ingredients and an average cooking time of 20 min including preparation). Recipe cards included instructions to produce the meals and nutritional facts and came with informational guidance about healthy meal planning. All were pretested before inclusion in the study (see Supplemental Material, for details).

#### Exercise Videos

Forty short exercise videos, each 2 min in length, were designed and filmed with input from one of the authors who is an expert in sport and exercise science. As the focus was on breaking up sedentary time, the videos were designed as simple exercises that could be performed easily from a desk or using home furniture (e.g., sofas) and were based on prior research showing the health benefits of such exercises (e.g., Carter & Gladwell, 2017; see Supplemental Material, for details). To guide their use of the videos, participants were given an information sheet about breaking up sitting time.

#### Meal and Movement Plans (For the Plan Condition Group)

Participants in the plan condition could select one of three different meal plans comprising two recipe cards a day (lunch and dinner) and one of three different movement plans with eight 2-min exercise videos to be completed each day.

#### Quantitative Measures

We collected quantitative measures via questionnaires sent to participants pre- and postintervention.

##### Primary Quantitative Outcomes

###### Usage of Study Resources

Participants indicated, poststudy only, the total number of times that they used the recipe cards and exercise videos on the platform during their study period.

###### Self-Efficacy

At two time points (pre- and poststudy), participants completed four items adapted from Linde et al. (2006) about whether they felt confident in performing a behavior under specific circumstances (e.g., “How confident are you that you would be able to eat healthily during the work week?”), measured on a scale of 0 (not at all confident) to 8 (extremely confident); Cronbach’s α = 0.7 (pre) & 0.74 (post).

##### Additional Quantitative Outcomes

We collected as secondary data participants’ reported pre- and poststudy levels of physical activity and attitudes toward exercise and healthy eating, and dietary quality during the study. We did not find evidence for differences between intervention groups on these secondary outcomes, and thus we report the measures and our analyses of them in the Supplemental Material.

#### Measures for Feasibility Analysis

We used a combination of qualitative and quantitative questions to assess the feasibility of the resources in the poststudy questionnaire. Participants indicated the factors that prevented them from using the recipe cards or exercise videos or the plans (for those who had one). Participants also indicated what factors they considered important to have in a plan. Participants could either select multiple options or provide their own text for these questions. In addition, participants who had a plan provided quantitative ratings of their attitudes to the plan in terms of ten adjectives (*annoying, interesting, credible, logical, easy to understand, personally relevant, confusing, complete, too long, useful*; Kothe & Mullan, 2014; Vandelanotte & De Bourdeaudhuij, 2003) measured on a 6-point Likert scale (1 = *strongly disagree* to 6 = *strongly agree*).

#### Qualitative Follow-Up Interviews

Participants indicated in their poststudy questionnaire if they consented to be contacted for a follow-up interview (details of participants who consented vs. those who did not are in Table 2). Participants who consented were randomly selected to be contacted by email. Interviews were held virtually using videoconferencing software and recorded with participants’ consent, then transcribed verbatim for subsequent analysis.

We used a semistructured interview format using a list of set questions for all participants, guided by the APEASE criteria (Affordability, Practicability, Effectiveness, Acceptability, Side effects/safety, Equity; see Table 3; Michie et al., 2014). The semistructured format allowed the interviewer to follow up on discussion points that participants identified (Brinkmann, 2014) and build rapport with participants (Barriball & While, 1994).[Table tbl3]

### Analytical Approach

#### Quantitative Analyses

##### Effects of the Plan Intervention

We used Bayesian analyses to compare the effect of the intervention condition (plan vs. control) on the use of recipe cards and exercise videos and on changes in self-efficacy. Bayesian analyses allow us to quantify the evidence that supports a model assuming an effect of the intervention condition relative to a model where no effect exists (i.e., the “null” model). By computing a “Bayes factor” (BF), we can observe the ratio between the likelihood of each of the models given the data (Wagenmakers et al., 2018). Crucially, this meant that we could quantify evidence for the null hypothesis (as opposed to frequentist inferences, whereby a *p* value > .05 indicates insufficient support for the effect, but not evidence of no effect; Dienes, 2014). Bayesian analyses are also more robust to changes in sample size because evidence is generated in favor of either model using posterior parameter estimates (Rouder, 2014). We implemented the Bayesian analyses in R using the BayesFactor package (Morey & Rouder, 2018).

Bayes factors are reported either as evidence for the hypothesized effect (BF_10_) or evidence against it (BF_01_). A BF_10_ of 10 indicates that the evidence for the hypothesized effect is ten times more likely than for no effect, whereas a BF_01_ of 10 indicates that the evidence for there being no effect is ten times more likely than for the hypothesized effect.

##### Effects of Self-Efficacy Beliefs

To assess whether participants’ self-efficacy beliefs affected their subsequent resource use, we conducted Bayesian bivariate correlations between prestudy self-efficacy for making healthy meals and taking short exercise breaks and use of recipe cards and exercise videos, respectively.

#### Feasibility Analyses

To understand the feasibility of the plan intervention and inform future practical implementation through web portal delivery, we conducted a descriptive analysis of the study feedback measures and a thematic analysis of the follow-up interviews. We also analyzed attitudes to the plans using a Bayesian general linear model regression that included the following predictors to assess their explanatory potential in shaping attitudes: use of resources, prestudy attitudes toward healthy eating and exercise, past exercise behavior age, gender, ethnicity, and proportion of time working from home.

#### Qualitative Analyses

The lead author conducted a thematic analysis using interpretive coding of each of the ten interview transcripts. We generally applied the reflexive approach to thematic analysis (Braun & Clarke, 2019), conducted by a single researcher and aiming to explore the diversity of interviewees’ experiences of the intervention. However, we also included elements of the “codebook” approach (Braun & Clarke, 2022) since we had some a priori expectations about elements relevant to intervention success. We therefore preregistered six themes that we expected to be deduced in the data. These themes reflected the APEASE criteria for evaluating behavioral change interventions (Michie et al., 2014; described in Table 3). Nonetheless qualitative data is rarely purely deductive (Braun & Clarke, 2012), and we also accommodated the induction of additional themes identified during the coding process. One additional “psychological” theme that did not clearly fit any of the preregistered themes (see Table 3) was identified this way.

Following the six-phase approach described in Braun and Clarke (2012), the researcher (a) familiarized herself with the data through repeated listening to the audio files and reading of the transcripts; (b) generated initial codes interpreting segments of the data relevant to the research question; (c) mapped the codes to the preregistered themes; (d) reviewed the themes and the coding subcategories within them, involving an iterative process where codes and their theme mappings were reviewed for appropriateness in the context of the entire data set; (e) defined and named the induced theme and codes within all themes to ensure that they were informative; and (f) produced the narrative report presenting the themes. The coded data set that resulted from Steps 1–5 and informed Step 6 is available on the OSF.

## Results

### Use of Resources (Recipe Cards and Exercise Videos) During the Study Period

Overall, participants reported using only 10%–20% of the number of recipe cards and exercise videos recommended for the week. Participants with a plan used the resources more than participants without them. Seventy-nine percent of participants with a plan used at least one recipe card over the week, as compared to 44% of participants in the control. On average, participants with a plan used more recipe cards (*M* = 3.09, *SD* = 1.50, 95% CI [2.18, 4.01]) than control participants (*M* = 1.15, *SD* = 1.50, 95% CI [0.62, 1.67]), BF_10_ = 77.67. For the exercise videos, 88% of participants with a plan used at least one, versus 76% in the control, and on average participants with a plan used a greater number of exercise videos (*M* = 9.73, *SD* = 9.68, 95% CI [6.30, 13.16]) than control participants (*M* = 3.65, *SD* = 6.48, 95% CI [1.39, 5.91]), BF_10_ = 10.82.

Self-reports may have inflated the number of resources used slightly, as the number of recipe card and exercise video loads from the web portal was lower than what participants reported. These data indicated evidence against an effect of the intervention plan on the number of recipe cards used, BF_01_ = 3.49, with, on average, control participants without a plan loading recipe cards only 0.41 times and participants with a plan loading recipe cards 0.55 times. However, follow-up interviews with participants indicated that they did take screenshots of the recipe cards or printed them for cooking. The record of how many times they loaded recipe cards from the site would therefore be lower overall compared to self-reported use if participants downloaded one and used it regularly offline. With the exercise videos, however, there remained strong evidence that participants with a plan used more exercise videos (*M* = 5.24, *SD* = 8.51) than those without a plan (*M* = 0.03, *SD* = 0.17), BF_10_ = 42.93—albeit less often than the self-reports.

### Self-Efficacy Did Not Predict Resource Use When There Was a Plan

There was slight evidence that participants’ self-reported self-efficacy prior to the study correlated positively with their self-reported use of the recipe cards, *r* = 0.25, 95% credible interval [0.03–0.45], BF_10_ = 2.68 and self-reported use of exercise videos, *r* = 0.24, 95% credible interval [0.01–0.45], BF_10_ = 2.50.^3^

Within the plan condition, we did not find evidence that self-efficacy was correlated with self-reported adherence to the planned recipe cards, *r* = 0.09, 95% CI [−0.23, 0.40], BF_01_ = 2.21, nor planned exercise videos, *r* = 0.10, 95% CI [−0.19, 0.43], BF_01_ = 1.82. Therefore, although participants with higher self-efficacy were in general more likely to use resources, when participants were given a plan their baseline levels of self-efficacy no longer predicted whether they were more likely to use the resources.

In contrast to our expectations, a paired-samples Bayesian *t* test showed that self-efficacy actually substantially decreased over the course of the study (see Table 3), BF_10_ = 91.05, *M*_change_ = −1.00, *SD* = 2.11, 95% CI [−1.51, −0.48]. An independent samples Bayesian *t* test on change in self-efficacy between conditions found inconclusive evidence, BF_10_ = 1.28.

### Feasibility of Web Resources

#### Barriers and Factors Important for Resource Use and Plans

**Figure 1 fig1:**
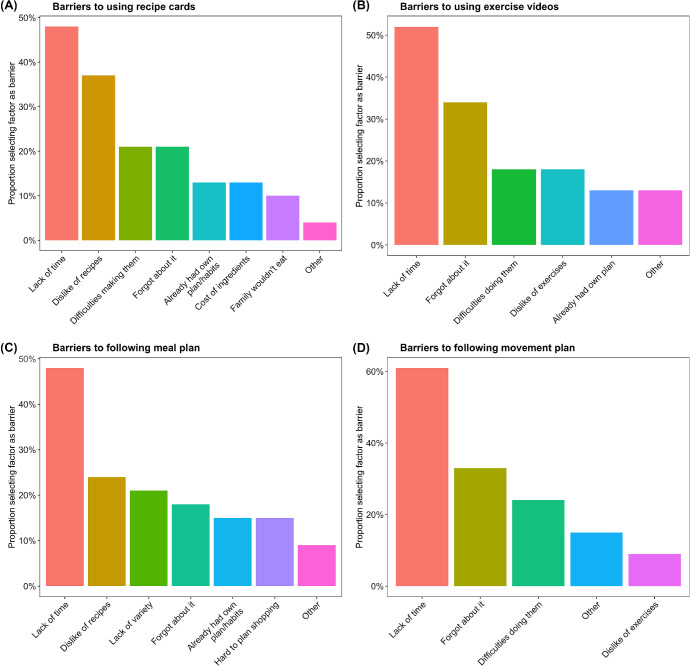
Reported Barriers to Using Recipe Cards (Top Left), Exercise Videos (Top Right), Meal Plans (Bottom Left), and Movement Plans (Bottom Right) *Note*. See the online article for the color version of this figure.

Figure 1 shows the proportion of respondents overall (top panels) and among the plan condition (bottom panels), who indicated they faced the respective barriers (on the *x*-axis) to using the recipe cards (top left), exercise videos (top right), and following the meal (bottom left) and movement plans (bottom right). Overwhelmingly, lack of time was the most frequently cited barrier by participants, with dislike of recipes being the next most frequent barrier for meal resources and plans, while forgetting to exercise was the next most frequent barrier for exercise resources and plans. Consistent with this, participants also ranked duration of cooking and exercise, respectively, as their most important factor to consider when creating a meal or movement plan, though interestingly, participants cited average preferred cooking and exercise times that were more than what they were offered in the study (30 and 19 min, respectively). Ease of performing the planned activities (cooking or exercising) was the next highest ranked factor on average.[Fig fig1]

#### Attitudes to Meal and Movement Plans

Overall, participants given a plan had slightly positive attitudes toward them (*M* = 4.50, *SD* = 0.60), with a bit more positivity for the movement (*M* = 4.73, *SD* = 0.66) than the meal plan (*M* = 4.27, *SD* = 0.74), BF_10_ = 4.11. We found moderate evidence that females felt more positively toward the plan than males, BF_10_ = 4.72, however, given the skewed gender sample, this result should not be interpreted conclusively. Overall, our exploratory Bayesian model indicated evidence *against* an effect of our predictor variables (adherence to the plan, prestudy attitudes and behavior, age, gender, ethnicity, and proportion of time working from home) on participants’ attitudes toward the plan, BF_01_ = 3.83.

### Qualitative Experience of Study: Thematic Analysis of Follow-Up Interviews

We were able to identify instances of all a priori themes from the transcripts, although they varied in frequency of occurrence (see Table 3). Practicability was the most frequent theme that most influenced the success of the intervention (found in all transcripts and on average mentioned 23 times per transcript). Side effects and safety (4 and 1, respectively) was the least frequent theme, where interviewees mainly brought up specific issues. We discuss each of the themes here with reference to the APEASE criteria and an emergent theme on psychological factors.

#### Affordability: Did the Resources Incur a Cost to Use?

In general, interviewees found that the exercise videos incurred no, or little, monetary cost, and this was conducive to using them. Opinions were more divided when it came to meals. Some interviewees perceived the suggested recipes as affordable and using ingredients they already had, however, others disagreed and perceived the recipes to be too costly:

A lot of these are outside the things that I normally buy, and times are hard … salmon … it looks really nice, um, but … it goes too far out of the normal [stuff] I have to buy. (P06)

#### Practicability: Were There Logistical or Knowledge Barriers to Using the Resources?

This was the most frequent theme that manifested in various subthemes of mainly logistical challenges (and how they were overcome). One logistical constraint was specific to using the meal resources, where interviewees largely cited the need to work around their family’s preferences and requirements as a barrier to using the recipe cards. Interviewees who had children found it was a particular challenge, “just factoring in everybody, in the house, that have so many different requirements” (P07). One interviewee acknowledged that a possible way to manage this was to introduce new recipes more gradually, rather than all at once in a plan.

The most common practical barrier was simply that life got in the way, resulting in interviewees being “engrossed and caught up in [their] work” (P09) and thus forgetting to use the exercises throughout the day. This was sometimes compounded by a lack of transition time between meetings, which also squeezed out intentions to follow an exercise video. However, interviewees felt that the short duration of the exercises was helpful in overcoming this challenge as they felt less like an interference with the working day.

Some interviewees who had a plan mentioned that the plan helped to combat the challenge of being too engrossed in work, as it “reminded you every day that you had to do it, um, gave a little bit of accountability” (P04). In contrast, interviewees who did not have a plan thought that having one would have helped, as:

It doesn’t cross my mind. So like, reminders to do something that’s short and doesn’t really impact your day too much would be beneficial. I know it really has in the past. (P07)

A facilitator in terms of improving the resources’ practicability was very much the simplicity of the resources, in particular the videos. Nearly, all interviewees commented on the low barrier to entry of using the resources as a positive factor. The simplicity of the recipes was also commented on, though this might not have been sufficient to overcome other barriers to their use. For this, a few physical logistics came up, mainly to do with organizing food shopping for the meals for some participants.

#### Effectiveness: Did Participants Perceive That the Resources Worked Well?

Interviewees brought up effectiveness in terms of their perceived benefits of the resources being motivation to use them—but also the lack thereof as a barrier. Experiencing direct physical benefits from using the exercises was associated with a positive feeling from being less sedentary, which was surprising for some interviewees who had not expected the short stints to “feel like it made a difference, and I wasn’t getting to the end of the day thinking, I sat in the same position for hours and hours on end” (P01).

Although interviewees often did not use the recipe cards due to other barriers, they mentioned that the information and ideas they gained had incidental impact in terms of influencing behavior for the future:

I think what the recipes made me realize, is actually they weren’t too complicated … because they were simple, and it made me realize that most of it was just stuff I already eat, but adding in kind of some extra healthy elements. (P09)

However, interviewees’ existing expectations did color whether they found the resources effective. One interviewee (P03) mentioned having higher expectations for meals, which made them unmotivated to use the recipes as they preferred to spend more time cooking than to use recipes they found uninspiring.

#### Acceptability: Did Participants Feel That Using the Resources Would Be Supported in Their Organization?

Organizational norms came up as a factor that would influence the use of resources. One aspect of this was the “top-down” nature of workplace norms, and the perceived need to have “permission to actually have, like, a two three-minute break” (P08).

Another aspect was what colleagues would think if one was seen exercising at work—even though this is unlikely when working from home. Changing perceived workplace norms around taking breaks might have wider effects on behavior, as one interviewee mentioned that if all their colleagues were to partake, it would ease the worry that one was slacking off by practicing healthy habits at work (P05).

#### Safety: Did Participants Worry About Using Resources Safely?

Safety was not a great concern among interviewees, and only came up in the context of the exercise videos. One particular issue they raised was the fact that some videos depicted employees using static chairs as props. One interviewee (P03) suggested that some additional instructions in the videos could be helpful, but on the whole, interviewees felt the exercise videos were sufficiently simple that they could safely perform them.

#### Equity: Did Interviewees Feel That Their Unique Personal Circumstances Acted as a Barrier to Use of the Resources?

Highlighting the importance of flexibility, most interviewees mentioned the need to work around their personal circumstances. Existing health issues was the most common personal circumstance raised, with six interviewees mentioning injuries or chronic issues. However, most participants felt that there was sufficient flexibility offered by the range of exercise videos available, that they were able to cater for their personal situation. This was the case even for interviewees who were suggested a specific set of exercises in their plan.

#### Emergent Theme: Psychological Factors That Facilitated or Prevented Effective Use of Resources

Two main psychological factors that influenced the use of resources were the prevalence of habits and participants’ perception of the resources—both of which could act in different ways. Participants acknowledged that existing habits played an important role in how much they were able to integrate the resources into their life, with the COVID-19 pandemic restrictions from the past 2 years in particular having had a detrimental effect on building habits that were then difficult to overturn. However, the impact of habits might be related to how participants perceived the resources. One interviewee who was habitually active felt the exercises were “eating into recovery time” for them, rather than something to be incorporated into the daily routine (P10). Other interviewees reflected that the resources had changed their perception of exercise:

I don’t think that I ever think about the fact that exercise can be done in less than a certain amount of time … so I thought it was really good that you could have these kinds of, like, snackable amounts of exercise. (P04)

The short, 2-min exercise videos were perceived to have longer term beneficial effects in shaping their views of how exercise could fit into their lives, particularly those who had negative perceptions of exercise to begin with. For example, one interviewee highlighted that the videos were “a big highlight for me because they didn’t make me feel bad about myself and my abilities” (P05), while another felt like 2-min stints were psychologically something they could stick to in the longer term, especially when it was part of a “movement plan” (instead of “exercise plan”; P07).

Psychological perception of how the resources were delivered also differed among interviewees. Most of the male interviewees mentioned a sense of psychological “overwhelm” that they experienced during the study because of the number of emails they were sent. On the other hand, this was not mentioned at all by the female interviewees, two of whom (with a plan) mentioned that the emails were facilitators rather than barriers for them.

## Discussion

Behavior change models such as COM-B propose that successfully converting intentions into action relies on a combination of factors, described as an individual’s capability, opportunity, and motivation to engage in the behavior (Michie et al., 2011). We investigated whether these factors were helpful to understand the extent to which desk-based employees who worked from home would engage with web-based self-help health resources to move more and produce more balanced meals. We found that our sample, despite being fairly confident of their ability to carry out these behaviors before they started the study, appeared to fall into an “intention-behavior gap” (Orbell & Sheeran, 1998; Sniehotta et al., 2005; Webb & Sheeran, 2006) and did not utilize the web resources to the full extent recommended. For example, participants completed only 10% of the recommended eight short exercises spaced throughout the day. However, certain factors appeared to encourage resource usage and engagement. We discuss these factors and their implications for successful intervention design in the context of the COM-B framework.

### Improve Capabilities: Plan in Simple Activities

The web-based resources were primarily targeted at increasing participants’ capability to break up their sitting time and prepare balanced meals. In the interviews, participants did perceive themselves to have capacity to perform the exercises, with their simplicity as a key factor that enabled them to fit them in without disrupting work. Health practitioners often stress the importance of simplicity and making the recommended actions easy to perform when promoting health behaviors (e.g., Koelen & Lindström, 2005; Michie et al., 2011). The plans we gave to half the participants provided more simplification of these participants’ actions, for example, by giving them access to relevant guidance at the time of action (Roy et al., 2022). Indeed, we observed that average exercise video use was at least three times higher in the plan than the no-plan condition—a finding that supports existing evidence for the effectiveness of action planning (Barz et al., 2016; Gollwitzer, 1999; Kwasnicka et al., 2013; Luszczynska et al., 2011). Critically, prestudy self-efficacy levels were related to overall engagement with resources during the study, but prestudy self-efficacy levels did not predict how much participants who had a plan adhered to it. This suggests that having a plan could be especially helpful in circumstances, where individuals may have initial reservations about their capability to perform the desired behavior.

Both the quantitative and qualitative analyses also showed that participants faced more barriers with the recipe cards than the exercise videos, which reflect the greater complexity inherent in preparing meals. Indeed, in designing the meal plans, the research team had to consider simplifying all stages of the process, from planning and shopping for ingredients to cooking, yet this ultimately did not overcome complexities such as family requirements and personal preferences and habits. As such, simplifying actions for making balanced meals may mean suggesting minimal adjustments to existing routines, rather than new actions that are simple to perform. An alternative could be to suggest “food swaps,” that is, simple adjustments to meals that individuals already intend to make, such as ways to increase vegetables or swapping out less healthy ingredients. This approach has had some success with changing food shopping or behavior (Breathnach et al., 2022; Jansen et al., 2021), so future research may wish to explore its feasibility in a home cooking context.

### Create Opportunities to Use Resources: Organizing Around Logistics of Life and Work

Lack of opportunity was a major barrier to intervention success: About half of our participants in our feasibility analyses cited lack of time as a barrier to using the self-help resources, and the theme that “life got in the way” clearly emerged among all our interviewees, who mentioned that their good intentions fell by the wayside once inundated with work (impeding exercise video use) or family commitments (impeding recipe card use).

Addressing the barriers to opportunity posed by these external factors may require different approaches for movement than meals. Interviewees frequently mentioned the pressure to work constantly without breaks, suggesting that changes to perceived workplace culture may be necessary to create opportunities for exercise breaks may require changes to perceived workplace culture.

Perspectives differed over where the pressure originated from: For one interviewee, it was a top-down pressure, for others, it was an internalized or a perceived norm of getting caught up in tasks or having little transition time between online meetings. Indeed, such norms seem to have permeated home-based working, contributing to fatigue (Collewet & Sauermann, 2017), sitting more (Meyer et al., 2020), and poorer eating habits (Robinson et al., 2021)—all of which harm productivity (Pencavel, 2015). The plan was cited as something that created opportunities to escape these working norms, especially as it came with a daily email to remind participants of their selected activities. Interestingly, participants without a plan also had these reminders, but would still have had to search for and select an exercise or recipe rather than clicking through to the predetermined one. It was thus not just the reminder that created the opportunity, but a reminder that also simplified the action to take. This is in line with previous work that found implementation intentions paired with text message reminders was more effective to prompt exercising than any of the two alone (Prestwich et al., 2009). Overall, it suggests that well-timed prompting could be another way to generate opportunities to take the exercise break as long as it works in tandem with other psychological components.

In contrast to exercises, meals were subject to greater family than work pressures, especially for participants who had children and/or struggled to persuade their families to change their eating habits. Planning which recipe cards to use helped slightly, but not as much as with exercises, possibly because participants’ shopping habits also varied a lot, which affected their opportunities to use the recipe cards during the study. Providing opportunities to try new recipes in a home-based context may require greater flexibility and better tailoring of the meal resources to the individual’s living situation (e.g., Eyles & Mhurchu, 2009) as compared to onsite interventions where employers have more control over what food is offered at the office. Given the complexities of meal preparation, it may be worth conceptualizing different steps of the process (e.g., shopping, cooking) as offering multiple opportunities to plan in small changes over time instead (e.g., adding one set of new ingredients at the next shopping day and then using those to introduce one new recipe in the week).

### Motivating Changes Through Changing Psychological Perspectives

The planning intervention was expected to tackle motivation by simplifying the cognitive control involved in directing healthier behaviors. This was especially effective for the movement plan. Interviewees described the plan as a “reminder” and something that “provided accountability” to complete their exercises, in line with our expectation that it would be a practical and psychological facilitator. Beyond planning, we also observed some potential longer term psychological benefits. Some interviewees mentioned changing their perspective on how easy it was to introduce more vegetables into their diet, while others redefined their previous concepts of exercise as hard and uncomfortable. These qualitative insights point to other considerations for supporting motivational aspects of behavior change, especially when introducing new behaviors that people may feel uncertain about. Using accessible language (e.g., “moving every hour”) could lower the initial perceived effort involved—reducing cognitive costs to initiating behavior as well as improving capabilities. Indeed, if people feel their goals are unachievable, it can negatively impact their motivation to continue (Nuss & Li, 2021; O’Keeffe et al., 2020). People may also respond to plans that can be updated over time to align with changes in their motivational needs. For instance, plans that last for short durations could motivate people to start, after which allowing people to reflect on their experience could prompt them to revise their preconceptions and positively update their capability and plans to perform the behavior in the future.

### Implications for Theory and Practice

Overall, the factors that facilitated or hindered the effectiveness of our intervention on healthier eating and breaking up sedentary time could each be related to the components posited by the COM-B model. We therefore derive two main contributions from our study toward existing theory and practice in the behavioral change domain. First, we found that often, a single factor addressed more than one component. For example, the simplicity of the exercises not only helped with the capability to perform them, but also with the psychological motivation to initiate them, which further strengthens capability; likewise for the simplicity offered by having a plan. Our findings thus provide evidence to support that elements of the theoretical COM-B model are linked (e.g., capability affects motivation), but we additionally posit that these interlinkages are bidirectional (e.g., motivation also affects capability) and could even act to reinforce each other over time.

Second, our study provides confirmatory evidence that the external context is an importance influence of behavior, even when one intends to exert control. Although the planning intervention was more successful overall than having no plan, it worked better for addressing sedentary behavior than dietary behavior, and still did not produce the ideal behavioral outcome. Planning seemed insufficient to overcome the situational barriers such as work and family demands that participants could not control. These barriers appear to go beyond participants’ perception of control over their behavior (as posited in the theory of planned behavior; Ajzen, 1991), since self-efficacy was no longer significantly related to resource use when participants had a plan. Rather, our results suggest that situational barriers are better conceptualized as a structural lack of opportunity to enact the plan—in this, we align with the COM-B model and its focus on opportunity as the “context” driving behaviors (Michie et al., 2011). Our intervention thus supports the relevance of using COM-B as a model to guide and tailor intervention design in the home-working setting, and it is important to align interventions with the way capability, opportunity, and motivation manifest for the specific behavior targeted.

### Limitations and Future Directions

Our study benefited from its mixed-methods approach, which allowed us to combine a feasibility analysis, qualitative data, and quantitative data from an embedded intervention to interpret these data as a whole, gain more insight about why and how planning helped, and evaluate the process of the intervention. However, there are several limitations that constrain our conclusions. First, the study was conducted with a small, self-selected sample from a single organization, which included a relatively large proportion of females. We therefore do not assume that it would automatically generalize to organizations in other industries with differing work roles. We can also infer that our self-selected sample had a desire to adopt the healthier behaviors, which was helpful for studying the intention-behavior gap, but organizations also include individuals who may not see the value of these behaviors, and might need different interventions to convince them of their importance.

Second, it only followed up with participants immediately after the trial, with a focus on whether the intervention could sustain engagement with self-help resources, so any longer term behavior changes or benefits effects on subsequent health or productivity at work—although prior research has indicated that engaging with resources to break up sedentary time is likely to have a positive impact (Carter & Gladwell, 2017). Further work is needed to expand our interventions to larger and more diverse samples and to ascertain if our initial findings can be scaled up and result in longer term impact on health and productivity.

Third, we report a reflexive thematic analysis of the follow-up interviews conducted by the first author. This interpretive approach emphasizes the reflections of the researcher in the identification of themes; as such, the interpretation of the data is subjective and there is the possibility that certain themes may have been unidentified, oversimplified or overidentified (Braun & Clarke, 2023).

Finally, although we found that having a plan was better than not having one, engagement with the resources on the whole was still not ideal relative to the recommendations of the study, especially with regards to recipe card use. Our feasibility analyses and qualitative interviews gave some insight on what facilitated engagement and what acted as a barrier, so future research could build on this study to further investigate whether more detailed aspects of planning (e.g., offering more personalization within a plan) or the frequency and channel of plan reminders (e.g., sending reminders multiple times a day, using emails or phone alerts) could increase engagement even further.

Overall, our study contributes to a small but growing body of work on interventions targeted at occupational health behavior change for employees working from home whose roles are largely sedentary. Our findings suggest that it is feasible to help employees plan in regular movements and (to a lesser extent) healthy meals when working from home, and having this plan would be more effective than simply giving employees the web resources to explore on their own. More work is still needed to raise engagement levels up to the recommended standard, but planning appears to be a helpful addition to organize self-help resources to be more useful to home workers who would like to use them.

## Supplementary Material

10.1037/ocp0000356.supp

## Figures and Tables

**Table 1 tbl1:** Demographic and Prestudy Characteristics of Sample, by Study Condition

Characteristic	No-plan (*n* = 34)	Plan (*n* = 33)	Overall (*n* = 67)
Ethnicity (proportion)			
White British	79%	79%	79%
White other	12%	18%	15%
Other races	9%	3%	6%
Median (mean) days per week working from home	5 (4.91)	5 (4.70)	5 (4.81)
Mean (*SD*) daily sedentary time in hours	7.14 (1.16)	7.37 (0.85)	7.25 (1.02)
Proportion in part time work	6%	9%	7%
Proportion with dietary restrictions^a^	12%	18%	15%
Proportion with food allergies	9%	9%	9%
Proportion of activity type (in past week)			
Active (≥150 min)	74%	82%	78%
Fairly active (30–149 min)	21%	15%	18%
Inactive (<30 min)	3%	6%	4%
Median (mean) minutes of exercise in past week^b^	280 (365)	360 (374)	315 (369)
Proportion reporting effortful exercise in past week	74%	64%	69%
*Note*. None of these characteristics were substantially different between conditions (for all comparisons, BF_01_ > 1.63 in a Bayesian independent samples *t* test and *p* > .150 in a conventional independent samples *t* test).			
^a^ Due to the resources available for this study, we only included participants who reported dietary needs that could be supported within the study (ovo-lacto vegetarian or gluten-free). ^b^ Total exercise in the past week was predominantly undertaken as walking (median = 240 min), with only a minority indicating cycling or sport, fitness and dance activities (median = 0 min for both).			

**Table 2 tbl2:** Details of Participants Who Consented (Or Not) to Being Contacted for Interview, and of the Final Interview Sample

Characteristic	Did not consent to follow up (*n* = 30)	Consented to follow up (*n* = 37)	Interviewed (*n* = 10/37 who consented)
Median (mean) days per week working from home	5 (4.80)	5 (4.81)	5 (4.77)
Mean (*SD*) hours sitting per day	7.26 (1.09)	7.25 (0.97)	7.22 (1.23)
No. in part time work	0	5	1
No. with dietary restrictions (ovo-lacto vegetarian or gluten-free)	3	7	3
No. with food allergies	3	3	1
Proportion of activity type (in past week)			
Active (≥150 min)	24	28	7
Fairly active (30–149 min)	5	7	1
Inactive (<30 min)	2	1	2
Median (mean) minutes of exercise in past week			
Prestudy	260 (367)	320 (371)	260 (317)
Poststudy	300 (362)	300 (383)	288 (334)
No. reporting effortful exercise in past week			
Prestudy	21	25	5
Poststudy	26	27	5
Mean (*SD*) attitude toward healthy eating			
Prestudy	5.35 (0.76)	5.16 (0.97)	4.73 (1.19)
Poststudy	5.45 (0.71)	5.46 (0.87)	4.99 (0.99)
Mean (*SD*) attitude toward exercise			
Prestudy	3.55 (0.64)	3.49 (0.80)	3.00 (0.77)
Poststudy	3.68 (0.58)	3.46 (0.96)	3.10 (1.05)
Mean (*SD*) self-efficacy			
Prestudy	5.43 (1.62)	5.38 (1.87)	4.98 (1.77)
Poststudy	4.12 (1.35)	4.64 (1.72)	3.90 (1.72)

**Table 3 tbl3:** Themes in the Qualitative Follow-Up Interviews

Theme	Definition	Indicative examples from transcripts	No. of transcripts including theme	Mean freq. within transcripts	
				Average count	Proportion of all coded mentions
Affordability	Whether the participant faced any monetary costs of following the intervention	“That’s purely down to things like cost. I can’t—the problem is a lot of these are outside the things that I normally buy, and times are hard so I’ve not been able to really look.” [P06]	6	1.6	2.5%
Practicability	Whether the participant faced logistical and/or knowledge barriers to using the resources in the intervention.	“it’s all pretty simple, you don’t even need, you don’t need anybody telling you what to do, it’s quite obvious what you need to do.” [P01]	10	23.3	37.9%
Effectiveness	How much the participant felt the intervention improved their diet and/or reduced their sedentary behavior.	“I thought they were all really good, and they weren’t too—they were enough to energies you and to kind of feel the hit, after the day, but they weren’t so much so that you were like sweating profusely.” [P04]	10	10.2	16.3%
Acceptability	Whether the participant supported the use of the intervention in the context of employee health and well-being services.	“Some of them I was like, oh, would I do that at my desk at work, my colleague is sat next to me, but I guess that’s just my personal, um (pause) embarrassment level.” [P04]	9	4.4	8.5%
Side effects/safety	Whether the participant had any safety concerns in undertaking any of the activities in the resources (e.g., performing certain exercises, using a recommended cooking technique)	“Ones where you’re sort of, er, doing, like squats, I didn’t do those ones because I didn’t want to put too much pressure, onto my spine.” [P08]	4	1.0	1.7%
Equity	Whether the participant felt that their unique personal circumstances prevented them from using the resources or prevented the resources from being effective for them specifically (as compared to others).	“I suppose the emphasis was on no this is quick sort of quick to do but, I don’t mind spending more time on something if it’s, if it’s going to be really good and I felt like, the recipes just needed to be a bit more, inspiring.” [P03]	10	6.6	11.1%
Psychological	Whether the participant mentioned psychological factors that facilitated or prevented their effective use of the resources.	“Only in that, I suppose psychologically, you think, oh, okay, this is doable, whereas maybe I wouldn’t have thought about it before.” [P07]	10	15.5	26.5%
*Note*. The first six themes were hypothesized a priori based on the APEASE criteria (Affordability, Practicability, Effectiveness, Acceptability, Side effects/safety, Equity; Michie et al., 2014) and deduced from codes in the data. The psychological theme was an emergent theme identified from bottom-up (inductive) coding of the data.					

## Footnotes

1 While there are no established guidelines yet for sitting time targets, adults are estimated to spend 58%–70% of their waking hours sedentary (Koster et al., 2012; Owen et al., 2010; Spittaels et al., 2012). During work hours, this proportion would be higher, with one study reporting that office workers spend 77% (or 6.6 hr) of their work time sitting, often in prolonged bouts (Thorp et al., 2012). We therefore used a conservative 60% of a typical 8-hr work day to determine our cutoff for eligibility.

2 The interview male/female gender ratio was balanced as we had planned to gain more insight into potential differences in experiences for both genders. Recruitment for interviews took place as soon as possible following each study period so that interviewees’ experiences would be relatively fresh in memory and also to meet funding-related deadlines for data collection. As such, we followed the original plan for a balanced gender interviewee ratio. However, as the final sample was predominantly female, this meant that the male interviewees captured a larger proportion of the male participants (50%) than the female interviewees (9%).

3 Analyses on the correlation between self-efficacy and site-recorded use of recipe cards and exercise videos was weaker, for recipe cards: BF_10_ = 1.25, *r* = 0.20, 95% CI [−0.03, 0.42]; for exercise videos: BF_10_ = 1.94, *r* = 0.23, 95% CI [0.0004, 0.43].

## References

[ref1] Abraham C., & Graham-Rowe E. (2009). Are worksite interventions effective in increasing physical activity? A systematic review and meta-analysis. Health Psychology Review, 3(1), 108–144. 10.1080/17437190903151096

[ref2] Ajzen I. (1991). The theory of planned behavior. Organizational Behavior and Human Decision Processes, 50(2), 179–211. 10.1016/0749-5978(91)90020-T

[ref3] Anderson L. M., Quinn T. A., Glanz K., Ramirez G., Kahwati L. C., Johnson D. B., Buchanan L. R., Archer W. R., Chattopadhyay S., Kalra G. P., Katz D. L., & the Task Force on Community Preventive Services. (2009). The effectiveness of worksite nutrition and physical activity interventions for controlling employee overweight and obesity: A systematic review. Systematic Reviews, 37(4), 340–357. 10.1016/j.amepre.2009.07.003 19765507

[ref4] Barriball K. L., & While A. (1994). Collecting data using a semi-structured interview: A discussion paper. Journal of Advanced Nursing, 19(2), 328–335. 10.1111/j.1365-2648.1994.tb01088.x 8188965

[ref5] Bartmann N., Cloughesy J. N., Probst B. M., Romagnoli G., & Woerner A. (2023). Behavioral Interventions to improve home-based office-workers’ health. Trends in Psychology, 31(1), 89–104. 10.1007/s43076-021-00122-x PMC8754544 40477899

[ref6] Barz M., Lange D., Parschau L., Lonsdale C., Knoll N., & Schwarzer R. (2016). Self-efficacy, planning, and preparatory behaviours as joint predictors of physical activity: A conditional process analysis. Psychology & Health, 31(1), 65–78. 10.1080/08870446.2015.1070157 26155825

[ref7] Bélanger-Gravel A., Godin G., & Amireault S. (2013). A meta-analytic review of the effect of implementation intentions on physical activity. Health Psychology Review, 7(1), 23–54. 10.1080/17437199.2011.560095 23005579

[ref8] Binyamin S. S., & Hoque M. R. (2020). Understanding the drivers of wearable health monitoring technology: An extension of the unified theory of acceptance and use of technology. Sustainability. 10.3390/su12229605

[ref9] Braun V., & Clarke V. (2012). Thematic analysis. American Psychological Association. 10.1037/13620-004

[ref10] Braun V., & Clarke V. (2019). Reflecting on reflexive thematic analysis. Qualitative Research in Sport, Exercise and Health, 11(4), 589–597. 10.1080/2159676X.2019.1628806

[ref11] Braun V., & Clarke V. (2022). Conceptual and design thinking for thematic analysis. Qualitative Psychology, 9(1), 3–26. 10.1037/qup0000196

[ref12] Braun V., & Clarke V. (2023). Toward good practice in thematic analysis: Avoiding common problems and be(com)ing a *knowing* researcher. International Journal of Transgender Health, 24(1), 1–6. 10.1080/26895269.2022.2129597 36713144 PMC9879167

[ref13] Breathnach S., Lally P., Llewellyn C. H., Sutherland A., & Koutoukidis D. A. (2022). Strategies to reduce the energy content of foods pre-ordered for lunch in the workplace: A randomised controlled trial in an experimental online canteen. The International Journal of Behavioral Nutrition and Physical Activity, 19(1), Article 54. 10.1186/s12966-022-01257-5 35550584 PMC9096740

[ref14] Brinkmann S. (2014). Unstructured and semi-structured interviewing. In Leavy P. (Ed.), The Oxford handbook of qualitative research (pp. 277–299). Oxford University Press.

[ref15] Carter S. E., & Gladwell V. F. (2017). Effect of breaking up sedentary time with callisthenics on endothelial function. Journal of Sports Sciences, 35(15), 1508–1514. 10.1080/02640414.2016.1223331 27559678

[ref16] Chavez-Dreyfuss G. (2020). The number of permanent remote workers is set to double in 2021. https://www.weforum.org/agenda/2020/10/permanent-remote-workers-pandemic-coronavirus-covid-19-work-home/

[ref17] Collewet M., & Sauermann J. (2017). Working hours and productivity. Labour Economics, 47, 96–106. 10.1016/j.labeco.2017.03.006

[ref18] Conner M., Rodgers W., & Murray T. (2007). Conscientiousness and the intention-behavior relationship: Predicting exercise behavior. Journal of Sport & Exercise Psychology, 29(4), 518–533. 10.1123/jsep.29.4.518 17968051

[ref19] Dienes Z. (2014). Using Bayes to get the most out of non-significant results. Frontiers in Psychology, 5, Article 781. 10.3389/fpsyg.2014.00781 25120503 PMC4114196

[ref20] Eyles H. C., & Mhurchu C. N. (2009). Does tailoring make a difference? A systematic review of the long-term effectiveness of tailored nutrition education for adults. Nutrition Reviews, 67(8), 464–480. 10.1111/j.1753-4887.2009.00219.x 19674343

[ref21] Eynon M., Foad J., Downey J., Bowmer Y., & Mills H. (2019). Assessing the psychosocial factors associated with adherence to exercise referral schemes: A systematic review. Scandinavian Journal of Medicine & Science in Sports, 29(5), 638–650. 10.1111/sms.13403 30742334

[ref22] Faghy M. A., Duncan M. J., Pringle A., Meharry J. B., & Roscoe C. M. P. (2022). UK university staff experience high levels of sedentary behaviour during work and leisure time. International Journal of Occupational Safety and Ergonomics, 28(2), 1104–1111. 10.1080/10803548.2021.1874704 33428548

[ref23] Fitzgerald S., Kirby A., Murphy A., & Geaney F. (2016). Obesity, diet quality and absenteeism in a working population. Public Health Nutrition, 19(18), 3287–3295. 10.1017/S1368980016001269 27230727 PMC5197930

[ref24] Goettler A., Grosse A., & Sonntag D. (2017). Productivity loss due to overweight and obesity: A systematic review of indirect costs. BMJ Open, 7(10), Article e014632. 10.1136/bmjopen-2016-014632 PMC5640019 28982806

[ref25] Gollwitzer P. M. (1999). Implementation intentions: Strong effects of simple plans. American Psychological Association. 10.1037/0003-066X.54.7.493

[ref26] Hall G., Laddu D. R., Phillips S. A., Lavie C. J., & Arena R. (2021). A tale of two pandemics: How will COVID-19 and global trends in physical inactivity and sedentary behavior affect one another? Progress in Cardiovascular Diseases, 64, 108–110. 10.1016/j.pcad.2020.04.005 32277997 PMC7194897

[ref27] Healy G., Winkler E., Dunstan D., Matthews C., & Owen N. (2010). Objectively measured sedentary time, physical activity and cardio-metabolic risk in adults: NHANES (USA) 2003–2006. Journal of Science and Medicine in Sport, 12, e203–e204. 10.1016/j.jsams.2009.10.424

[ref28] Hendren S., & Logomarsino J. (2017). Impact of worksite cafeteria interventions on fruit and vegetable consumption in adults: A systematic review. International Journal of Workplace Health Management, 10(2), 134–152. 10.1108/IJWHM-12-2016-0089

[ref29] Jansen L., van Kleef E., & Van Loo E. J. (2021). The use of food swaps to encourage healthier online food choices: A randomized controlled trial. The International Journal of Behavioral Nutrition and Physical Activity, 18(1), Article 156. 10.1186/s12966-021-01222-8 34863208 PMC8642761

[ref30] Koelen M. A., & Lindström B. (2005). Making healthy choices easy choices: The role of empowerment. European Journal of Clinical Nutrition, 59(1), S10–S16. 10.1038/sj.ejcn.1602168 16052175

[ref31] Koster A., Caserotti P., Patel K. V., Matthews C. E., Berrigan D., Van Domelen D. R., Brychta R. J., Chen K. Y., & Harris T. B. (2012). Association of sedentary time with mortality independent of moderate to vigorous physical activity. PLOS ONE, 7(6), Article e37696. 10.1371/journal.pone.0037696 22719846 PMC3374810

[ref32] Kothe E. J., & Mullan B. A. (2014). Acceptability of a theory of planned behaviour email-based nutrition intervention. Health Promotion International, 29(1), 81–90. 10.1093/heapro/das043 22942273

[ref33] Kwasnicka D., Presseau J., White M., & Sniehotta F. F. (2013). Does planning how to cope with anticipated barriers facilitate health-related behaviour change? A systematic review. Health Psychology Review, 7(2), 129–145. 10.1080/17437199.2013.766832

[ref34] Linde J. A., Rothman A. J., Baldwin A. S., & Jeffery R. W. (2006). The impact of self-efficacy on behavior change and weight change among overweight participants in a weight loss trial. American Psychological Association. 10.1037/0278-6133.25.3.282 16719599

[ref35] Luszczynska A., Schwarzer R., Lippke S., & Mazurkiewicz M. (2011). Self-efficacy as a moderator of the planning-behaviour relationship in interventions designed to promote physical activity. Psychology & Health, 26(2), 151–166. 10.1080/08870446.2011.531571 21318927

[ref36] Macridis S., Johnston N., Johnson S., & Vallance J. K. (2018). Consumer physical activity tracking device ownership and use among a population-based sample of adults. PLOS ONE, 13(1), Article e0189298. 10.1371/journal.pone.0189298 29293532 PMC5749689

[ref37] Marcus B. H., King T. K., Clark M. M., Pinto B. M., & Bock B. C. (1996). Theories and techniques for promoting physical activity behaviours. Sports Medicine, 22, 321–331. 10.2165/00007256-199622050-00005 8923649

[ref38] Mattioli A. V., Sciomer S., Cocchi C., Maffei S., & Gallina S. (2020). Quarantine during COVID-19 outbreak: Changes in diet and physical activity increase the risk of cardiovascular disease. Nutrition, Metabolism, and Cardiovascular Diseases, 30(9), 1409–1417. 10.1016/j.numecd.2020.05.020 PMC7260516 32571612

[ref39] McDermott M. S., Oliver M., Simnadis T., Beck E. J., Coltman T., Iverson D., Caputi P., & Sharma R. (2015). The Theory of Planned Behaviour and dietary patterns: A systematic review and meta-analysis. Preventive Medicine, 81, 150–156. 10.1016/j.ypmed.2015.08.020 26348455

[ref40] McEachan R. R. C., Conner M., Taylor N. J., & Lawton R. J. (2011). Prospective prediction of health-related behaviours with the theory of planned behaviour: A meta-analysis. Health Psychology Review, 5(2), 97–144. 10.1080/17437199.2010.521684

[ref41] Meyer J., McDowell C., Lansing J., Brower C., Smith L., Tully M., & Herring M. (2020). Changes in physical activity and sedentary behavior in response to covid-19 and their associations with mental health in 3052 US adults. International Journal of Environmental Research and Public Health, 17(18), Article 6469. 10.3390/ijerph17186469 32899495 PMC7559240

[ref42] Michie S., Abraham C., Whittington C., McAteer J., & Gupta S. (2009). Effective techniques in healthy eating and physical activity interventions: A meta-regression. Health Psychology, 28(6), 690–701. 10.1037/a0016136 19916637

[ref43] Michie S., Atkins L., & West R. (2014). The behaviour change wheel: A guide to designing interventions (1st ed.). Silverback Publishing.

[ref44] Michie S., Johnston M., & Carey R. (2020). Behavior change techniques. In Gellman M. D. (Ed.), Encyclopedia of behavioral medicine (pp. 206–213). Springer International Publishing.

[ref45] Michie S., Johnston M., Francis J., Hardeman W., & Eccles M. (2008). From theory to intervention: Mapping theoretically derived behavioural determinants to behaviour change techniques. Applied Psychology, 57(4), 660–680. 10.1111/j.1464-0597.2008.00341.x

[ref46] Michie S., van Stralen M. M., & West R. (2011). The behaviour change wheel: A new method for characterising and designing behaviour change interventions. Implementation Science, 6(1), Article 42. 10.1186/1748-5908-6-42 21513547 PMC3096582

[ref47] Morey R. D., & Rouder J. N. (2018). BayesFactor: Computation of bayes factors for common designs. https://richarddmorey.github.io/BayesFactor/

[ref48] Munir F., Biddle S. J. H., Davies M. J., Dunstan D., Esliger D., Gray L. J., Jackson B. R., O’Connell S. E., Yates T., & Edwardson C. L. (2018). Stand More AT Work (SMArT Work): Using the behaviour change wheel to develop an intervention to reduce sitting time in the workplace. BMC Public Health, 18(1), Article 319. 10.1186/s12889-018-5187-1 29510715 PMC5840779

[ref49] Nuss K., & Li K. (2021). Motivation for physical activity and physical activity engagement in current and former wearable fitness tracker users: A mixed-methods examination. Computers in Human Behavior, 121, Article 106798. 10.1016/j.chb.2021.106798

[ref50] O’Keeffe N., Scheid J. L., & West S. L. (2020). Sedentary behavior and the use of wearable technology: An editorial. International Journal of Environmental Research and Public Health, 17(12), Article 4181. 10.3390/ijerph17124181 32545429 PMC7345036

[ref51] Olander E. K., Fletcher H., Williams S., Atkinson L., Turner A., & French D. P. (2013). What are the most effective techniques in changing obese individuals’ physical activity self-efficacy and behaviour: A systematic review and meta-analysis. The International Journal of Behavioral Nutrition and Physical Activity, 10(1), Article 29. 10.1186/1479-5868-10-29 23452345 PMC3639155

[ref52] Oni T., Micklesfield L. K., Wadende P., Obonyo C. O., Woodcock J., Mogo E. R. I., Odunitan-Wayas F. A., Assah F., Tatah L., Foley L., Mapa-Tassou C., Bhagtani D., Weimann A., Mba C., Unwin N., Brugulat-Panés A., Hofman K. J., Smith J., Tulloch-Reid M., … Wareham N. J. (2020). Implications of COVID-19 control measures for diet and physical activity, and lessons for addressing other pandemics facing rapidly urbanising countries. Global Health Action, 13(1), Article 1810415. 10.1080/16549716.2020.1810415 32867606 PMC7480567

[ref53] Orbell S., & Sheeran P. (1998). “Inclined abstainers”: A problem for predicting health-related behaviour. British Journal of Social Psychology, 37(2), 151–165. 10.1111/j.2044-8309.1998.tb01162.x 9639861

[ref54] Owen N., Sparling P. B., Healy G. N., Dunstan D. W., & Matthews C. E. (2010). Sedentary behavior: Emerging evidence for a new health risk. Mayo Clinic Proceedings, 85(12), 1138–1141. 10.4065/mcp.2010.0444 21123641 PMC2996155

[ref55] Parekh N., & Deierlein A. L. (2020). Health behaviours during the coronavirus disease 2019 pandemic: Implications for obesity. Public Health Nutrition, 23(17), 3121–3125. 10.1017/S1368980020003031 32746955 PMC7522472

[ref56] Pencavel J. (2015). The productivity of working hours. Economic Journal, 125(589), 2052–2076. 10.1111/ecoj.12166

[ref57] Peters S. E., Dennerlein J. T., Wagner G. R., & Sorensen G. (2022). Work and worker health in the post-pandemic world: A public health perspective. The Lancet Public Health, 7(2), e188–e194. 10.1016/S2468-2667(21)00259-0 35122760 PMC8809900

[ref58] Prestwich A., Perugini M., & Hurling R. (2009). Can the effects of implementation intentions on exercise be enhanced using text messages? Psychology & Health, 24(6), 677–687. 10.1080/08870440802040715 20205020

[ref59] Public Health England. (2018). Plans to cut excess calorie consumption unveiled. https://www.gov.uk/government/news/plans-to-cut-excess-calorie-consumption-unveiled

[ref60] Ráthonyi G., Kósa K., Bács Z., Ráthonyi-Ódor K., Füzesi I., Lengyel P., & Bácsné Bába É. (2021). Changes in workers’ physical activity and sedentary behavior during the COVID-19 pandemic. Sustainability, 13(17), Article 9524. 10.3390/su13179524

[ref61] Richter A. (2020). Do privacy concerns prevent employees’ acceptance of smart wearables and collaborative robots? In Reinhardt D., Langweg H., Witt B. C., & Fischer M. (Eds.), SICHERHEIT 2020 (pp. 141–146). Gesellschaft für Informatik e.V. 10.18420/sicherheit2020_14

[ref62] Robinson E., Boyland E., Chisholm A., Harrold J., Maloney N. G., Marty L., Mead B. R., Noonan R., & Hardman C. A. (2021). Obesity, eating behavior and physical activity during COVID-19 lockdown: A study of UK adults. Appetite, 156, Article 104853. 10.1016/j.appet.2020.104853 33038479 PMC7540284

[ref63] Rouder J. N. (2014). Optional stopping: No problem for Bayesians. Psychonomic Bulletin & Review, 21(2), 301–308. 10.3758/s13423-014-0595-4 24659049

[ref64] Roy D., Berry E., & Dempster M. (2022). “If it is not made easy for me, I will just not bother”. A qualitative exploration of the barriers and facilitators to recycling plastics. PLOS ONE, 17(5), Article e0267284. 10.1371/journal.pone.0267284 35503782 PMC9064103

[ref65] Sniehotta F. F., Scholz U., & Schwarzer R. (2005). Bridging the intention–behaviour gap: Planning, self-efficacy, and action control in the adoption and maintenance of physical exercise. Psychology & Health, 20(2), 143–160. 10.1080/08870440512331317670

[ref66] Spittaels H., Van Cauwenberghe E., Verbestel V., De Meester F., Van Dyck D., Verloigne M., Haerens L., Deforche B., Cardon G., & De Bourdeaudhuij I. (2012). Objectively measured sedentary time and physical activity time across the lifespan: A cross-sectional study in four age groups. The International Journal of Behavioral Nutrition and Physical Activity, 9(1), Article 149. 10.1186/1479-5868-9-149 23249449 PMC3542099

[ref67] Thorp A. A., Healy G. N., Winkler E., Clark B. K., Gardiner P. A., Owen N., & Dunstan D. W. (2012). Prolonged sedentary time and physical activity in workplace and non-work contexts: A cross-sectional study of office, customer service and call centre employees. The International Journal of Behavioral Nutrition and Physical Activity, 9(1), Article 128. 10.1186/1479-5868-9-128 23101767 PMC3546308

[ref68] Tuman M., & Moyer A. (2019). Health intentions and behaviors of health app owners: A cross-sectional study. Psychology Health and Medicine, 24(7), 819–826. 10.1080/13548506.2019.1576911 30729803

[ref69] Vandelanotte C., & De Bourdeaudhuij I. (2003). Acceptability and feasibility of a computer-tailored physical activity intervention using stages of change: Project FAITH. Health Education Research, 18(3), 304–317. 10.1093/her/cyf027 12828232

[ref70] Verplanken B., & Faes S. (1999). Good intentions, bad habits, and effects of forming implementation intentions on healthy eating. Wiley. 10.1002/(SICI)1099-0992(199908/09)29:5/6<591::AID-EJSP948>3.0.CO;2-H

[ref71] Wagenmakers E.-J., Marsman M., Jamil T., Ly A., Verhagen J., Love J., Selker R., Gronau Q. F., Šmíra M., Epskamp S., Matzke D., Rouder J. N., & Morey R. D. (2018). Bayesian inference for psychology. Part I: Theoretical advantages and practical ramifications. Psychonomic Bulletin & Review, 25(1), 35–57. 10.3758/s13423-017-1343-3 28779455 PMC5862936

[ref72] Webb T. L., & Sheeran P. (2006). Does changing behavioral intentions engender behavior change? A meta-analysis of the experimental evidence. Psychological Bulletin, 132(2), 249–268. 10.1037/0033-2909.132.2.249 16536643

[ref73] Xie J., Wen D., Liang L., Jia Y., Gao L., & Lei J. (2018). Evaluating the validity of current mainstream wearable devices in fitness tracking under various physical activities: Comparative study. JMIR mHealth and uHealth, 6(4), Article e94. 10.2196/mhealth.9754 29650506 PMC5920198

