# Supplementary material for: Planning Engagement With Web Resources to Improve Diet Quality and Break Up Sedentary Time for Home-Working Employees: A Mixed Methods Study
Source: J Occup Health Psychol. 2023 Aug;28(4):224–38. doi: 10.1037/ocp0000356 (PMC10424491; doi:10.1037/ocp0000356)
Supplement: Supplementary file 1 [file OCP-2022-0991_Supplementary_Materials.docx]

**Supplementary Information**

**Detailed study procedure**

The study lasted five days (Monday to Friday) for each participant, to cover one full work week. Participants signed up to the study at least ten days before their study period started to enable materials to be sent in advance. Participants gave informed consent and completed an initial questionnaire (items are described below) including pre-study measures as part of the sign-up process. As part of the pre-study questionnaire, we also collected demographic information about age, gender, ethnicity, working patterns (number of days working from home, full or part time work, time spent sitting), caring responsibilities, and dietary requirements. Thereafter, participants were randomised to the plan or no-plan control condition, and those in the plan condition selected one of three prepared plans for their meals and one of three prepared plans for daily movements. All participants received a confirmation email with a reminder of their selected study period. For participants in the plan condition, the confirmation email included their selected plans.

Ten days prior to the study period, participants were sent a free membership login to the KFEF web portal^[[1]](#footnote-1)^. This enabled them to familiarise themselves with the platform. Four days prior to the study period, participants received a study email that contained information in PDF formats, and a reminder of the selected plans for participants in the plan condition. The PDF information included a recommended shopping list, guide to meal planning, and advice sheet on breaking up sitting time. Sending this information in advance allowed participants to make any preparations and purchases needed before the study began.

**Detailed explanation of materials**

We focused on two types of self-help resources in this study: recipe cards and exercise videos to support balanced diets and reduce sedentary time respectively. This section explains our rationale for this focus.

**Recipe cards to help with balanced diets.** A set of 30 recipes were designed by the second author (who is a trained dietician and expert in nutrition), with the objective of providing a balanced contribution to advised dietary targets (e.g., Public Health England, 2018). While it is difficult to ascertain a single, global measure of diet quality (Alkerwi, 2014), the World Health Organization and many other national health authorities offer some general guidance as to what it should comprise (World Health Organization, 2020). These include recommendations for the different levels of food types and nutrients that should be included in the diet, and often involve minimum portions for other food types and nutrients (e.g., at least five portions of fruit and vegetables per day), and maximum portions for others (e.g., reducing portions of sugars, fat, and salt). Studies have shown that these recommendations are associated with reduced risk of non-communicable disease (Mozaffarian et al., 2014; Te Morenga et al., 2014; World Health Organization, 2003) and obesity (Food and Agriculture Organization of the United Nations, 2010; Hooper et al., 2015). However, public knowledge of dietary guidelines is typically poor (Bookari et al., 2017; Brown et al., 2011), so it may be difficult for people to translate guidelines into practice when selecting meals. Therefore, the recipe cards were aimed at making it easy to create meals that were in accordance with the recommended dietary guidelines. The nutritional content of all recipes were displayed on the recipe cards.

To ensure that meals were indeed easy to prepare, all recipes had to fulfil two requirements: they had to contain no more than seven common, easily accessible main ingredients and require an average cooking time of 20 minutes including preparation. All recipes were tested by the first author and another volunteer not from the authorship team. Both have basic cooking skills and are not professionally trained in cooking, diet, or nutrition. Only recipes that met the estimated time target by both testers were retained for the study. The longest recipe took 25 minutes to prepare, and the shortest 15 minutes.

To check that recipes would not impose excessive food shopping costs to participants, we purchased all items on the recommended shopping list from a local supermarket in the same region as the organisation prior to the study. The cost of items is available on the OSF (<https://osf.io/dbk6z/?view_only=dc648d2d5fac4b57bf973624416051c6>). These costs (roughly £60-£70) were comparable to the average weekly food expenditure in the UK (£69.20, based on data available for the nearest period to the study dates; Office for National Statistics, 2022).

To accommodate differences in food preferences and tolerances, the recipes included recommendations for substitutions for common allergens (e.g., substituting seeds for nuts) and included within the set ten meals suitable for ovo-lacto vegetarians and ten for gluten-intolerant individuals^^[[2]](#footnote-2)^^.

**Exercise videos to break up sitting time.** Forty short exercise videos, each two minutes in length, were designed and filmed with input from one of the authors who is an expert in sport and exercise science. The focus was on breaking up sitting time, which is a major contributor to sedentary behaviour due to the accumulation of prolonged, uninterrupted periods spent sitting (Hadgraft et al., 2016; Thivel et al., 2018). Such long periods of uninterrupted sitting can occur even when individuals meet physical activity targets (e.g., exercising for 30 minutes followed by 8 hours of sitting; Thivel et al., 2018) and is a problem for office-based workers, who regularly sit for periods of longer than an hour (Ryan et al., 2011; Thorp et al., 2012). Therefore, the aim of the exercise videos was to make it simple to break up one’s sitting time, so as to benefit individuals regardless of their physical activity levels.

Breaking up sitting time with short bouts of activity (e.g., 2min walk in a 20min period) has been shown to benefit cardiometabolic health (Bailey & Locke, 2015), but activity that takes one away from the desk is not always feasible for office workers (Carter et al., 2015). With this in mind, short calisthenic exercises such as body weight squats and lunges are a good solution for breaking up sitting time, as they can be performed in small spaces without equipment. Short 2-minute bouts of these exercises were found to increase energy expenditure and improve vascular function in the legs and arms when used to disrupt sedentary periods compared to no breaks, or other interventions such as standing and walking breaks (Carter et al., 2015; Carter & Gladwell, 2017). The videos were therefore designed as simple exercises that could be performed easily from a desk or using home furniture (e.g., sofas). The exercises worked a variety of muscle groups and incorporated elements of strength, balance, and flexibility. Participants were also given an information sheet about how to break up sitting time, intended to help guide their use of the exercise videos (available on the OSF: <https://osf.io/dbk6z/?view_only=dc648d2d5fac4b57bf973624416051c6>).

**Planning intervention**

Participants in the plan condition selected at the sign-up phase a meal plan comprising two recipe cards a day (lunch and dinner) and a movement plan with eight two-minute exercise videos to be completed each day. These plans put together combinations of the recipe cards and exercise videos. When selecting their plan, participants could choose either a general (no dietary restrictions), ovo-lacto vegetarian, or gluten-free meal plan, and a movement plan where videos focused on exercising at the desk, from a sofa, or a mixture of both. All plans are available on the OSF.

**Detailed description of primary quantitative outcomes**

Quantitative measures were administered to participants before and after the intervention as a questionnaire.

**Usage of study resources.** We used participants’ self-reported use of the study resources as the primary outcome measure in the study. Participants indicated during the post-study questionnaire the total number of times that they used the recipe cards and exercise videos on the platform during their study period. In addition, the KFEF platform also provided web-capture data on the number of times participants loaded these materials during the study period^^[[3]](#footnote-3)^^.

**Self-efficacy.** We include a measure of self-efficacy to assess whether the intervention would affect or interact with this measure in engaging in the desired behaviours. At two time points (pre- and post-study), participants indicated their confidence in undertaking healthy behaviours using a scale measure with four items adapted from Linde et al. (2006) about whether they felt confident in performing a behaviour under specific circumstances (e.g., “*How confident are you that you would be able to eat healthily during the work week?*”). This was measured on a scale of 0 (not at all confident) to 8 (extremely confident); Cronbach’s $\alpha$ = 0.7 (pre) & 0.74 (post).

**Secondary quantitative outcomes**

To assess if the intervention had any immediate impact on activity levels and diet quality, participants completed pre- and post-study measures of physical activity and attitudes towards exercise and healthy eating as part of the same questionnaire as the primary quantitative outcomes. They also completed at three time points during the study a checklist of foods consumed.

**Short Active Lives Survey (SALS).** The SALS is a tool recommended by Sport England to measure frequency, duration, and intensity of three types of common activities (walking, cycling, and sport, fitness and dance) in the past seven days and determine an individuals’ total weekly moderate physical activity (Sport England, 2021). Participants completed this as an indicator of past exercise behaviour.

**Attitudes towards exercise.** Participants rated their agreement with four concepts about exercise: difficult/easy, relaxing/stressful, not enjoyable/enjoyable, unhealthy/healthy (Poobalan, Aucott, Clarke, & Smith, 2012), measured on a five-point Likert scale (1: disagree to 5: agree); Cronbach’s $\alpha$ = 0.73 (pre) & 0.77 (post).

**Attitudes towards healthy eating.** Participants completed the healthy eating motivation scale (Naughton, McCarthy, & McCarthy, 2015), assessed through agreement with seven questions about healthy eating (e.g., “*It is important to me that the food I eat keeps me healthy*”), measured on a seven-point Likert scale (1: strongly disagree to 7: strongly agree); Cronbach’s $\alpha$ = 0.83 (pre) & 0.73 (post).

**Daily Food Checklist.** On three separate days of the study, participants completed a simple checklist of foods they had eaten, adapted from the NIH Daily Food List (National Cancer Institute, 2020). Participants placed a tick for each time that they ate a food type in a separate meal or snack in the day. This short (~5 minute) checklist format was selected to reduce response burden (thus increasing data quality and reducing study attrition) and have less impact on one’s behaviour as it is less intrusive (Thompson et al., 2002). This measure allowed us to assess participants’ overall dietary quality.

**Secondary analyses: Effects of the plan intervention on diet, activity, and attitudes**

We conducted exploratory analyses on the effects of the plan condition on participants’ diet (as measured by the average intake of each food category in the Daily Food Checklist), changes in reported activity levels on the SALS (as measured by change in total activity time—frequency x duration), and changes in attitudes towards healthy eating and exercise. For each of these variables, we ran a Bayesian independent samples t-test with condition (plan or control) as the between-subjects variable.

Table S1 reports the changes in the study measures between the pre- and post-study time points. Our exploratory analyses did not find any evidence that any of the measures changed after the study, nor that the plan had an effect on changes in attitudes and behaviours or on diet quality during the study, BF_01_ > 1 for all measures.

Table S1

Mean (and standard deviation) levels of self-report variables pre- and post-study, by condition

|  | Condition: Plan | | Condition: No plan | |
| --- | --- | --- | --- | --- |
|  | **Pre-study** | **Post-study** | **Pre-study** | **Post-study** |
| Attitude to healthy eating | 5.27 (0.99) | 5.43 (0.88) | 5.23 (1.30) | 5.47 (1.29) |
| Attitude to exercise | 3.54 (1.00) | 3.54 (1.20) | 3.50 (1.05) | 3.57 (1.11) |
| Average self-efficacy | 5.65 (2.65) | 4.15 (2.31) | 5.15 (2.16) | 4.65 (2.17) |
| Minutes of exercise in past week | 387 (381) | 356 (288) | 352 (353) | 391 (342) |
| Proportion reporting effortful exercise in past week | 64% | 76% | 74% | 82% |

**References**

Alkerwi, A. (2014). Diet quality concept. *Nutrition*, *30*(6), 613–618. https://doi.org/https://doi.org/10.1016/j.nut.2013.10.001

Bailey, D. P., & Locke, C. D. (2015). Breaking up prolonged sitting with light-intensity walking improves postprandial glycemia, but breaking up sitting with standing does not. *Journal of Science and Medicine in Sport*, *18*(3), 294–298. https://doi.org/10.1016/j.jsams.2014.03.008

Bookari, K., Yeatman, H., & Williamson, M. (2017). Falling short of dietary guidelines – What do Australian pregnant women really know? A cross sectional study. *Women and Birth*, *30*(1), 9–17. https://doi.org/https://doi.org/10.1016/j.wombi.2016.05.010

British Nutrition Foundation. (2018). *Wheat intolerance and coeliac disease*. https://www.nutrition.org.uk/nutritionscience/allergy/wheat-intolerance-and-coeliac-disease.html

British Nutrition Foundation. (2020). *Majority unlikely to go plant-based in the New Year, BNF survey reveals*. https://www.nutrition.org.uk/press-office/pressreleases/plant-based.html

Brown, K. A., Timotijevic, L., Barnett, J., Shepherd, R., Lähteenmäki, L., & Raats, M. M. (2011). A review of consumer awareness, understanding and use of food-based dietary guidelines. *British Journal of Nutrition*, *106*(1), 15–26. https://doi.org/DOI: 10.1017/S0007114511000250

Carter, S. E., & Gladwell, V. F. (2017). Effect of breaking up sedentary time with callisthenics on endothelial function. *Journal of Sports Sciences*, *35*(15), 1508–1514. https://doi.org/10.1080/02640414.2016.1223331

Carter, S. E., Jones, M., & Gladwell, V. F. (2015). Energy expenditure and heart rate response to breaking up sedentary time with three different physical activity interventions. *Nutrition, Metabolism and Cardiovascular Diseases*, *25*, 503–509. https://doi.org/10.1016/j.numecd.2015.02.006

Food and Agriculture Organization of the United Nations. (2010). *Fats and fatty acids in human nutrition: report of an expert consultation*. <https://www.who.int/nutrition/publications/nutrientrequirements/fatsandfattyacids_humannutrition/en/>

Hadgraft, N. T., Healy, G. N., Owen, N., Winkler, E. A. H., Lynch, B. M., Sethi, P., Eakin, E. G., Moodie, M., LaMontagne, A. D., Wiesner, G., Willenberg, L., & Dunstan, D. W. (2016). Office workers’ objectively assessed total and prolonged sitting time: Individual-level correlates and worksite variations. *Preventive Medicine Reports*, *4*, 184–191. https://doi.org/https://doi.org/10.1016/j.pmedr.2016.06.011

Hooper, L., Abdelhamid, A., Bunn, D., Brown, T., Summerbell, C. D., & Skeaff, C. M. (2015). Effects of total fat intake on body weight. *Cochrane Database of Systematic Reviews*, *8*, 1465–1858. https://doi.org/10.1002/14651858.CD011834

Linde, J. A., Rothman, A. J., Baldwin, A. S., & Jeffery, R. W. (2006). The impact of self-efficacy on behavior change and weight change among overweight participants in a weight loss trial. American Psychological Association. <https://doi.org/10.1037/0278-6133.25.3.282>

Mozaffarian, D., Fahimi, S., Singh, G. M., Micha, R., Khatibzadeh, S., Engell, R. E., Lim, S., Danaei, G., Ezzati, M., & Powles, J. (2014). Global Sodium Consumption and Death from Cardiovascular Causes. *New England Journal of Medicine*, *371*, 624–634. https://doi.org/10.1056/nejmoa1304127

National Cancer Institute. (2020). *Daily food checklist*. https://epi.grants.cancer.gov/dietary-assessment/daily.html

Naughton, P., McCarthy, S. N., & McCarthy, M. B. (2015). The creation of a healthy eating motivation score and its association with food choice and physical activity in a cross sectional sample of Irish adults. *International Journal of Behavioral Nutrition and Physical Activity*, *12*(74), 1–10. https://doi.org/10.1186/s12966-015-0234-0

Office for National Statistics, UK (2022). Family spending in the UK: April 2020 to March 2021. <https://www.ons.gov.uk/peoplepopulationandcommunity/personalandhouseholdfinances/expenditure/bulletins/familyspendingintheuk/april2020tomarch2021#family-spending-in-the-uk>

Poobalan, A. S., Aucott, L. S., Clarke, A., & Smith, W. C. S. (2012). Physical activity attitudes, intentions and behaviour among 18–25 year olds: A mixed method study. *BMC Public Health*, *12*(1), 640. https://doi.org/10.1186/1471-2458-12-640

Public Health England. (2018). *Plans to cut excess calorie consumption unveiled*. UK Government. <https://www.gov.uk/government/news/plans-to-cut-excess-calorie-consumption-unveiled>

Ryan, C. G., Dall, P. M., Granat, M. H., & Grant, P. M. (2011). Sitting patterns at work: objective measurement of adherence to current recommendations. *Ergonomics*, *54*(6), 531–538. https://doi.org/10.1080/00140139.2011.570458

Sport England. (2021). *Active Lives*. <https://www.sportengland.org/know-your-audience/data/active-lives?section=measures->

Te Morenga, L. A., Howatson, A. J., Jones, R. M., & Mann, J. (2014). Dietary sugars and cardiometabolic risk: systematic review and meta-analyses of randomized controlled trials of the effects on blood pressure and lipids. *The American Journal of Clinical Nutrition*, *100*(1), 65–79. https://doi.org/10.3945/ajcn.113.081521

Thivel, D., Tremblay, A., Genin, P. M., Panahi, S., Rivière, D., & Duclos, M. (2018). Physical Activity, Inactivity, and Sedentary Behaviors: Definitions and Implications in Occupational Health. *Frontiers in Public Health*, *6*, 288. https://doi.org/10.3389/fpubh.2018.00288

Thompson, F. E., Subar, A. F., Brown, C. C., Smith, A. F., Sharbaugh, C. O., Jobe, J. B., Mittl, B., Gibson, J. T., & Ziegler, R. G. (2002). Cognitive research enhances accuracy of food frequency questionnaire reports: Results of an experimental validation study. *Journal of the American Dietetic Association*, *102*(2), 212–225. https://doi.org/10.1016/S0002-8223(02)90050-7

Thorp, A. A., Healy, G. N., Winkler, E., Clark, B. K., Gardiner, P. A., Owen, N., & Dunstan, D. W. (2012). Prolonged sedentary time and physical activity in workplace and non-work contexts: a cross-sectional study of office, customer service and call centre employees. *International Journal of Behavioral Nutrition and Physical Activity*, *9*(1), 128. https://doi.org/10.1186/1479-5868-9-128

World Health Organization. (2003). *Diet, nutrition and the prevention of chronic diseases: report of a Joint WHO/FAO Expert Consultation. WHO Technical Report Series, No. 916*. https://www.who.int/dietphysicalactivity/publications/trs916/en/

World Health Organization. (2020). *Healthy diet*. https://www.who.int/news-room/fact-sheets/detail/healthy-diet

1. The web portal offered resources within seven “modules”: physical activity, healthy eating, fitness in pregnancy, mental health training, sleep hygiene, financial planning, and retirement planning. Participants were free to use any resources during the study, but received emails directing them towards the physical activity and healthy eating modules. [↑](#footnote-ref-1)
2. These dietary restrictions account for the two most common dietary restrictions in the UK (British Nutrition Foundation, 2018, 2020). While random allocation of a set of meals to three different plans would provide the best randomised control, this would also increase the level of non-adherence due to dietary restrictions. We thus sought to be inclusive of at least these two common dietary restrictions. [↑](#footnote-ref-2)
3. We had initially planned to track the number of minutes spent viewing these materials on the platform. However, based on qualitative interviews with participants, during which they discussed that the ways they used the materials could be offline (e.g., taking a screenshot to facilitate using a recipe card while cooking), we recognised that number of downloads was a more sensible measure of materials usage. [↑](#footnote-ref-3)
